# Correction: LC-MS analysis and hypotensive effect *via* inhabiting GLUT 1 and activating NO/Akt/eNOS signalling pathway of *Myrica rubra*

**DOI:** 10.1039/d2ra90053j

**Published:** 2022-05-27

**Authors:** Jing Li, Huiling Wang, Jian Li, Yonggang Liu, Hong Ding

**Affiliations:** Key Laboratory of Combinatorial Biosynthesis and Drug Discovery, Ministry of Education, School of Pharmaceutical Sciences, Wuhan University Wuhan China

## Abstract

Correction for LC-MS analysis and hypotensive effect *via* inhabiting GLUT 1 and activating NO/Akt/eNOS signalling pathway of *Myrica rubra* by Hong D. *et al. RSC Adv.*, 2020, **10**, 5371–5384, https://doi.org/10.1039/C9RA05895H.

The authors regret that an incorrect version of Fig. 7 was included in the original article. The correct version of Fig. 7 is presented below.

**Fig. 7:**
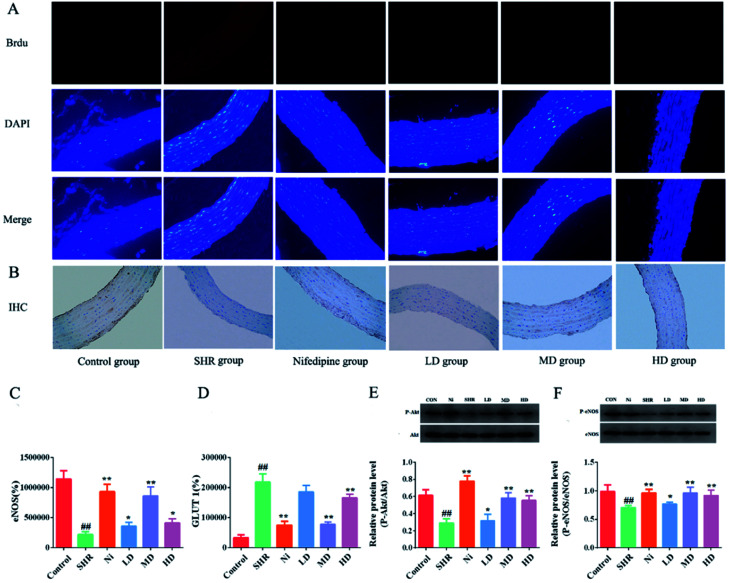
Analysis determining the expression of phosphorylation of GLUT 1, eNOS, and Akt in vascular endothelium. (A) Immunofluorescence. (B) Immunohistochemical analysis. (C) Optical density of GLUT 1 immunofluorescence. (D) Area of eNOS immunohistochemistry. The levels of phosphorylation eNOS and total eNOS proteins were quantified by densitometry. (E) The levels of phosphorylation Akt and total Akt proteins were quantified by densitometry (F). Each value represents mean ± SEM and the significance accepted at ^##^ indicates *p* < 0.01, compared with the respective normal control group. * and ** indicate *p* < 0.05 and *p* < 0.01, respectively, compared with the SHR group.

The Royal Society of Chemistry apologises for these errors and any consequent inconvenience to authors and readers.

## Supplementary Material

